# Risk reduction of hospitalisation and severe disease in vaccinated COVID-19 cases during the SARS-CoV-2 variant Omicron BA.1-predominant period, Navarre, Spain, January to March 2022

**DOI:** 10.2807/1560-7917.ES.2023.28.5.2200337

**Published:** 2023-02-02

**Authors:** Iván Martínez-Baz, Camino Trobajo-Sanmartín, Ana Miqueleiz, Itziar Casado, Ana Navascués, Cristina Burgui, Carmen Ezpeleta, Jesús Castilla, Marcela Guevara, the Working Group for the Study of COVID-19 in Navarra, Members of the Working Group for the Study of COVID-19 in Navarra, Miguel Fernández-Huerta, Carmen Martín, María Eugenia Portillo, Ingrid Estévez, Igberto Tordoya, Delia Quílez, Francisco Lameiro, Ana Isabel Álvaro, Eva Ardanaz, Fernando Baigorria, Aurelio Barricarte, Nerea Egüés, Manuel García Cenoz, Nerea Iriarte, Conchi Moreno-Iribas, Carmen Sayón

**Affiliations:** 1 Instituto de Salud Pública de Navarra, Pamplona, Spain; 2 CIBER Epidemiología y Salud Pública (CIBERESP), Pamplona, Spain; 3 Navarra Institute for Health Research (IdiSNA), Pamplona, Spain; 4 Clinical Microbiology Department, Hospital Universitario de Navarra, Pamplona, Spain; 5 Additional members of the Working Group for the Study of COVID-19 in Navarra are listed under Collaborators

**Keywords:** COVID-19, SARS-CoV-2, COVID-19 vaccine, Omicron variant, COVID-19 hospitalisation, severe COVID-19

## Abstract

**Background:**

As COVID-19 vaccine effectiveness against SARS-CoV-2 infection was lower for cases of the Omicron vs the Delta variant, understanding the effect of vaccination in reducing risk of hospitalisation and severe disease among COVID-19 cases is crucial.

**Aim:**

To evaluate risk reduction of hospitalisation and severe disease in vaccinated COVID-19 cases during the Omicron BA.1-predominant period in Navarre, Spain.

**Methods:**

A case-to-case comparison included COVID-19 epidemiological surveillance data in adults ≥ 18 years from 3 January–20 March 2022. COVID-19 vaccination status was compared between hospitalised and non-hospitalised cases, and between severe (intensive care unit admission or death) and non-severe cases using logistic regression models.

**Results:**

Among 58,952 COVID-19 cases, 565 (1.0%) were hospitalised and 156 (0.3%) were severe. The risk of hospitalisation was reduced within the first 6 months after full COVID-19 vaccination (complete primary series) (adjusted odds ratio (aOR): 0.06; 95% CI: 0.04–0.09) and after 6 months (aOR: 0.16; 95% CI: 0.12–0.21; p_comparison_ < 0.001), as well as after a booster dose (aOR: 0.06: 95% CI: 0.04–0.07). Similarly, the risk of severe disease was reduced (aOR: 0.13, 0.18, and 0.06, respectively). Compared with cases fully vaccinated 6 months or more before a positive test, those who had received a booster dose had lower risk of hospitalisation (aOR: 0.38; 95% CI: 0.28–0.52) and severe disease (aOR: 0.38; 95% CI: 0.21–0.68).

**Conclusions:**

Full COVID-19 vaccination greatly reduced the risk of hospitalisation and severe outcomes in COVID-19 cases with the Omicron variant, and a booster dose improved this effect in people aged over 65 years.

**Key public health message**

**What did you want to address in this study?**

A high incidence of COVID-19 in vaccinated people was observed during the SARS-CoV-2 Omicron variant circulation period. We aimed to evaluate if COVID-19 cases had lower risk of being hospitalised or presenting severe disease when vaccinated. We also wished to know if the booster dose of COVID-19 vaccine reduced the risk of hospitalisation and severe disease for COVID-19 in fully vaccinated individuals.

**What have we learnt from this study?**

Full COVID-19 vaccination reduced the risk of hospitalisation and severe outcomes in COVID-19 cases infected with the Omicron BA.1 variant. A booster dose notably improved this effect in older people. However, the benefit of a booster dose was not observed in adults up to 65 years in the early Omicron period.

**What are the implications of your findings for public health?**

Given the reduced effectiveness of the COVID-19 vaccines in preventing infection by the Omicron BA.1 variant, full COVID-19 vaccination of the population and the booster dose in the older people has been essential in reducing the incidence of severe COVID-19.

## Introduction

In November 2021, the World Health Organization designated a new severe acute respiratory syndrome coronavirus 2 (SARS-CoV-2) variant of concern as Omicron (Phylogenetic Assignment of Named Global Outbreak (Pango) lineage designation B.1.1.529) [1]. This variant caused a high incidence of coronavirus disease (COVID-19) cases in most European countries in early 2022 [2,3]. Several genetically related subvariants of Omicron have been detected. BA.1 was initially the predominant Omicron variant worldwide. By the end of February 2022, sequences designated as BA.2 progressively replaced BA.1 as the predominant Omicron variant in several countries, including Spain [4].

In Spain, the COVID-19 vaccination campaign, which began in December 2020, was successively targeted to adults from older to younger age groups [5] with the following vaccines: Comirnaty (BNT162b2 mRNA; BioNTech-Pfizer), Spikevax (mRNA-1273; Moderna), Vaxzevria (ChAdOx1 nCoV-19; Oxford-AstraZeneca) and Janssen vaccine (Ad26.COV2-S; Janssen-Cilag International NV) [6-9], with the majority (~84%) vaccinated with an mRNA vaccine [10]. From October 2021, a booster dose was progressively offered to fully vaccinated people starting with the oldest age group (≥ 80 years) and those immunocompromised, from 5 months after completing primary immunisation [5]. At the time when Omicron emerged, the vaccination coverage in Spain was 89.3% for the completed primary series in people older than 11 years and 62.3% for booster doses in people 70 years and older [10].

In countries with high COVID-19 vaccination coverage, an increasing proportion of SARS-CoV-2 infections was detected since December 2021 among vaccinated individuals [11]. Early reports suggested reduced COVID-19 vaccine effectiveness against infection caused by the SARS-CoV-2 Omicron vs the Delta (Pango lineage designation B.1.617.2) variant [12,13]. In addition, a booster dose appeared to provide only moderate extra protection against infection by the Omicron variant [14-17]. Thus, knowledge of the extent that COVID-19 vaccination reduces the risk of hospitalisation and severe disease among COVID-19 cases is important.

The present study aimed to evaluate the risk reduction of hospitalisation and severe disease in vaccinated COVID-19 cases compared with unvaccinated cases, and in fully vaccinated COVID-19 cases with a booster dose compared with those without a booster in Navarre, Spain, during the Omicron BA.1-predominant period.

## Methods

### Study setting, design and data sources

This study was performed in the region of Navarre (650,000 inhabitants), Spain, where the Navarre Health Service provides healthcare to residents, free of charge at the point of use. The study was conducted between 3 January and 20 March 2022, the weeks during which the Omicron variant was detected in more than 90% of cases tested for SARS-CoV-2 variant. The study included all people 18 years and older residing in Navarre who had a positive test for SARS-CoV-2 for the first time during the study period.

During the study period, there was high availability of free diagnostic tests and of personnel for testing of all suspected cases and close contacts of confirmed cases. Although healthcare professionals performed most tests, antigen tests were also available in pharmacies for self-testing and these results were also considered.

As part of the epidemiological surveillance, medical doctors reviewed all hospital admissions, intensive care unit (ICU) admissions and deaths of patients with confirmed SARS-CoV-2 infection during the 3 months after the positive test. A case-to-case comparison of COVID-19 vaccination status between hospitalised and non-hospitalised COVID-19 cases was conducted with individual data from the population-based cohort of COVID-19 confirmed cases from the enhanced epidemiological surveillance [18]. Information from SARS-CoV-2 test results (PCR, antigen test and self-testing), vaccination registry and electronic medical records related to the same individual was linked using a unique personal identifier. COVID-19 cases with a prior SARS-CoV-2 positive test and cases less than 18 years of age were excluded, as well as healthcare workers and nursing home residents because they have a different access to diagnostic tests and use of the healthcare system.

### Case definitions and SARS-CoV-2 testing 

 COVID-19 cases were confirmed for SARS-CoV-2 infection by quantitative reverse-transcription PCR (RT-qPCR) or antigen test in nasopharynx samples. COVID-19 hospitalised cases were defined as patients admitted for 24 h or more because of COVID-19, or who died from COVID-19. Deaths of confirmed COVID-19 cases that occurred outside the hospital were also considered. Severe disease included confirmed cases admitted to the ICU or who died from COVID-19. 

COVID-19 cases with PCR cycle threshold values ≤ 30 were further analysed by TaqPath COVID-19 RT-PCR kit (ThermoFisher Scientific) to get an approximation of the SARS-CoV-2 variant. The Omicron BA.1 subvariant contains the spike ΔH69/ΔV70 deletion, which is associated with spike gene target failure by TaqPath and was used as a proxy measure of this variant. The Omicron BA.2 subvariant does not contain the spike deletion and therefore is spike gene-positive [19,20].

### COVID-19 vaccination and covariables

The number and type of COVID-19 vaccine doses and date of administration were obtained from the regional vaccination register. We considered a case ‘fully vaccinated’ after receiving the complete primary vaccination series specified for each vaccine brand, i.e. one dose of Janssen vaccine or two doses of Comirnaty, Spikevax or Vaxzevria, and ‘partially vaccinated’ if only one of the two recommended doses of COVID-19 vaccine was received. An additional dose after the complete primary series was considered a booster dose. Each dose was considered potentially effective 14 days after administration. 

Study variables included age, sex, underlying conditions (immunocompromised, other major chronic conditions, and none), COVID-19 vaccination status, and month. Diagnoses of underlying conditions were obtained from electronic medical records of primary healthcare and included: liver cirrhosis, diabetes, cardiovascular diseases, cancer, chronic kidney disease, immunodeficiency, chronic obstructive pulmonary diseases, asthma, dementia, stroke, rheumatic diseases, and body mass index ≥ 40 kg/m^2^.

### Statistical analysis

We performed two main analyses. First, the COVID-19 vaccination status was compared between hospitalised and non-hospitalised COVID-19 cases. The second analysis compared the COVID-19 vaccination status between severe and non-severe COVID-19 cases. Five categories were considered for vaccination status: (i) unvaccinated as the reference category, (ii) partially vaccinated, (iii) fully vaccinated in the first 6 months before a positive test, (iv) fully vaccinated more than 6 months before a positive test and (v) fully vaccinated with a booster dose. 

The chi-squared test was used to compare proportions. Logistic regression models were employed to derive crude and adjusted odds ratio (aOR) with their 95% confidence intervals (CI) as a relative estimate of the risk reduction following vaccination. Adjusted models included age groups (18–44, 45–64, 65–74, 75–84 and ≥ 85 years), sex, underlying conditions (immunocompromised, other major chronic conditions and none) and month of positive test. The adjusted model was also used to compare the risk reduction associated with full vaccination within the first 6 months and 6 or more months after the last dose of vaccine.

Stratified analyses were carried out by age groups, sex, and presence of underlying conditions (yes/no). Additionally, a comparison was performed between COVID-19 cases fully vaccinated more than 6 months before a positive test and cases fully vaccinated with a booster dose. Sensitivity analyses were performed limited to weeks with more than 90% of the Omicron BA.1 subvariant (3 January–20 February 2022) and excluding self-testing to rule out possible bias because of these results.

All *statistical* analyses *were performed* with IBM SPSS Statistics 25 version (IBM, SPSS Inc.).

## Results

### Characteristics of COVID-19 cases

The study included 58,952 COVID-19 cases; 565 (1.0%) were hospitalised for COVID-19, and 156 (0.3%) were severe COVID-19 cases (39 of whom were admitted to the ICU and 126 died, with 9 meeting both criteria). Although 16.1% of cases were 65 years and older, they represented 71.9% of hospitalised and 80.1% of severe cases. Unvaccinated people accounted for 6.6% of cases, 26.5% of hospitalisations and 26.3% of severe cases. The proportion of hospitalisation and severe disease increased with age and the presence of underlying conditions (Table 1).

**Table 1:** Characteristics of the study population of COVID-19 cases by hospitalisation and severity status, Navarre, Spain, 3 January–20 March 2022 (n = 58,952)

Characteristics	COVID-19 cases							
	All n = 58,952		Hospitalised n = 565			Severe n = 156		
	n	%	n	%	p value^a^	n	%	p value^b^
**Age groups (years) **								
18–44	29,167	49.5	35	6.2	< 0.001	5	3.2	< 0.001
45–64	20,269	34.4	124	21.9		26	16.7	
65–74	4,797	8.1	91	16.1		25	16.0	
75–84	3,031	5.1	125	22.1		35	22.4	
≥ 85	1,688	2.9	190	33.6		65	41.7	
**Sex**								
Men	27,322	46.3	307	54.3	< 0.001	90	57.7	0.004
Women	31,630	53.7	258	45.7		66	42.3	
**Underlying conditions**								
Immunocompromised	501	0.8	28	5.0	< 0.001	11	7.1	< 0.001
Other major chronic conditions^c^	17,452	29.6	422	74.7		120	76.9	
None	40,999	69.5	115	20.4		25	16.0	
**COVID-19 vaccination status**								
Unvaccinated	3,873	6.6	150	26.5	< 0.001	41	26.3	< 0.001
Partially vaccinated	550	0.9	4	0.7		2	1.3	
Fully vaccinated < 6 months	25,662	43.5	25	4.4		9	5.8	
Fully vaccinated ≥ 6 months	10,467	17.8	86	15.2		21	13.5	
Fully vaccinated + booster dose	18,400	31.2	300	53.1		83	53.2	
**Month of positive test**								
Jan	46,553	79.0	429	75.9	< 0.001	123	78.8	0.107
Feb	8,398	14.2	113	20.0		28	17.9	
Mar	4,001	6.8	23	4.1		5	3.2	
**Period of Omicron variant circulation**								
3 Jan–20 Feb (> 90% of BA.1)	53,617	91.0	523	92.6	0.178	149	95.5	0.047
21 Feb–20 Mar (mix of BA.1 and BA.2)	5,335	9.0	42	7.4		7	4.5	
**Type of SARS-CoV-2 test**								
RT-qPCR	4,743	8.0	188	33.3	< 0.001	55	35.3	< 0.001
Antigen test	50,068	84.9	364	64.4		97	62.2	
Antigen self-testing	4,141	7.0	13	2.3		4	2.6	

COVID-19: coronavirus disease; RT-qPCR: reverse transcriptase quantitative PCR; SARS-CoV-2: severe acute respiratory syndrome coronavirus 2.

^a^ Chi-squared comparison between hospitalised and non-hospitalised cases.

^b^ Chi-squared comparison between severe and non-severe cases. P values < 0.05 were considered statistically significant.

^c^ Liver cirrhosis, diabetes, cardiovascular diseases, cancer, chronic kidney disease, chronic obstructive pulmonary diseases, asthma, dementia, stroke, rheumatic diseases, and body mass index ≥ 40 kg/m^2^.

A person was considered ‘fully vaccinated’ after receiving one dose of Janssen vaccine or two doses of Comirnaty, Spikevax or Vaxzevria, and ‘partially vaccinated’ if only one of the two recommended doses of COVID-19 vaccine received.

Unvaccinated COVID-19 cases were more frequently younger adults and had fewer underlying conditions. However, they were more likely admitted to the hospital or presented with severe disease. Supplementary Table S1 provides an overview of the study population by vaccination status.

A representative sample of 2,628 (4.5%) COVID-19 cases were tested with specific RT-qPCR tests to determine the SARS-CoV-2 variant and 2,575 (98.0%) were identified as the Omicron variant (n = 1,827 cases with the BA.1 subvariant and n = 748 with the BA.2 subvariant). Most cases included in this analysis (91.0%) were diagnosed between 3 January and 20 February 2021, when the subvariant Omicron BA.1 was predominant.

### Risk reduction of hospitalisation in vaccinated COVID-19 cases

The proportion of COVID-19 cases hospitalised was 3.9% among unvaccinated COVID-19 cases and 29.2% among unvaccinated cases aged 65 and older (Table 2). In the overall analysis, the risk of hospitalisation was reduced within the first 6 months of full COVID-19 vaccination (aOR: 0.06; 95% CI: 0.04–0.09) and after the sixth month (aOR: 0.16; 95% CI: 0.12–0.21; p_comparison_ < 0.001), as well as after a booster dose (aOR: 0.06; 95% CI: 0.04–0.07). Among fully vaccinated cases aged 65 years and older, risk reduction of hospitalisation seemed less pronounced after the sixth month of the last dose, although it was not statistically significant (aOR: 0.22 vs aOR: 0.11; p_comparison_ = 0.114). However, a booster dose was associated with a reduction in the risk of hospitalisation (aOR: 0.05; 95% CI: 0.04–0.07). The vaccination status had a similar effect in reducing the risk of hospitalisation regardless of sex, age and the presence of underlying conditions (Table 2).

**Table 2:** Risk reduction of hospitalisation in vaccinated COVID-19 cases vs unvaccinated cases by age, sex and underlying conditions, Navarre, Spain, 3 January–20 March 2022 (n = 58,952)

Study groups by vaccination status	COVID-19 cases			Crude analysis						Adjusted analysis^a^			
	All	Hospitalised		OR					95% CI	OR		95% CI	
	n	n	%										
**All cases **													
Unvaccinated	3,873	150	3.9	Reference						Reference			
Partially vaccinated	550	4	0.7	0.18					0.07–0.49	0.26		0.09–0.74	
Fully vaccinated < 6 months before positive test	25,662	25	0.1	0.02					0.02–0.04	0.06		0.04–0.09^b^	
Fully vaccinated ≥ 6 months before positive test	10,467	86	0.8	0.21					0.16–0.27	0.16		0.12–0.21^b^	
Fully vaccinated + booster dose	18,400	300	1.6	0.41					0.34–0.50	0.06		0.04–0.07	
**Aged 18–64 years **													
Unvaccinated	3,578	64	1.8	Reference						Reference			
Partially vaccinated	529	2	0.4	0.21		0.05–0.85				0.23		0.06–0.95	
Fully vaccinated < 6 months before positive test	25,453	18	0.1	0.04		0.02–0.07				0.05		0.03–0.08^b^	
Fully vaccinated ≥ 6 months before positive test	10,069	38	0.4	0.21		0.14–0.31				0.12		0.08–0.18^b^	
Fully vaccinated + booster dose	9,807	37	0.4	0.21		0.14–0.31				0.12		0.08–0.18	
**Aged ≥ 65 years **													
Unvaccinated	295	86	29.2	Reference						Reference			
Partially vaccinated	21	2	9.5	0.26			0.06–1.12			0.32	0.07–1.47		
Fully vaccinated < 6 months before positive test	209	7	3.3	0.08			0.04–0.19			0.11	0.05–0.26		
Fully vaccinated ≥ 6 months before positive test	398	48	12.1	0.33			0.23–0.49			0.22	0.14–0.34		
Fully vaccinated + booster dose	8,593	263	3.1	0.08			0.06–0.10			0.05	0.04–0.07		
**Men **													
Unvaccinated	1,820	74	4.1	Reference						Reference			
Partially vaccinated	257	3	1.2	0.28				0.09–0.89		0.44		0.13–1.47	
Fully vaccinated < 6 months before positive test	12,448	12	0.1	0.02				0.01–0.04		0.05		0.03–0.10^b^	
Fully vaccinated ≥ 6 months before positive test	4,878	44	0.9	0.22				0.15–0.31		0.14		0.10–0.22^b^	
Fully vaccinated + booster dose	7,919	174	2.2	0.53				0.40–0.70		0.06		0.04–0.09	
**Women **													
Unvaccinated	2,053	76	3.7	Reference						Reference			
Partially vaccinated	293	1	0.3	0.09			0.01–0.64			0.11		0.02–0.87	
Fully vaccinated < 6 months before positive test	13,214	13	0.1	0.03			0.01–0.05			0.06		0.03–0.12^b^	
Fully vaccinated ≥ 6 months before positive test	5,589	42	0.8	0.20			0.14–0.29			0.17		0.11–0.26^b^	
Fully vaccinated + booster dose	10,481	126	1.2	0.32			0.24–0.42			0.05		0.03–0.07	
**Cases with underlying conditions **													
Unvaccinated	930	95	10.2	Reference						Reference			
Partially vaccinated	113	4	3.5	0.32	0.12–0.90					0.49			0.17–1.44
Fully vaccinated < 6 months before positive test	5,280	15	0.3	0.03	0.01–0.04					0.07			0.04–0.13^b^
Fully vaccinated ≥ 6 months before positive test	3,002	67	2.2	0.20	0.15–0.28					0.20			0.14–0.28^b^
Fully vaccinated + booster dose	8,628	269	3.1	0.28	0.22–0.36					0.07			0.05–0.10
**Cases without underlying conditions**													
Unvaccinated	2,943	55	1.9	Reference						Reference			
Partially vaccinated	437	0	0.0	NA						NA			
Fully vaccinated < 6 months before positive test	20,382	10	0.0	0.03		0.01–0.05				0.04		0.02–0.08^b^	
Fully vaccinated ≥ 6 months before positive test	7,465	19	0.3	0.13		(0.08–0.23				0.10		0.06–0.17^b^	
Fully vaccinated + booster dose	9,772	31	0.3	0.17		0.11–0.26				0.03		0.02–0.06	

CI: confidence interval; COVID-19: coronavirus disease; NA: not available; OR: odds ratio.

^a^ OR adjusted for age (18–44, 45–64, 65–74, 75–84 and ≥ 85 years), sex, underlying conditions (immunocompromised, other major chronic conditions and none) and month.

^b^ Comparison within the logistic regression model between fully vaccinated cases ≥ 6 months and < 6 months before a positive COVID-19 test, p value < 0.05.

A person was considered ‘fully vaccinated’ after receiving one dose of Janssen vaccine or two doses of Comirnaty, Spikevax or Vaxzevria, and ‘partially vaccinated’ if only one of the two recommended doses of COVID-19 vaccine received.

Compared with cases fully vaccinated 6 months or more before a positive test, those who had received the booster dose had a significantly lower risk of hospitalisation (aOR:  0.38; 95% CI: 0.28–0.52) overall, and specifically among cases aged 65 years and older (aOR: 0.22; 95% CI: 0.16–0.32). However, the booster dose did not reduce the risk of hospitalisation of fully vaccinated people aged 18–64 years (aOR: 0.95; 95% CI: 0.58–1.53) (Table 3).

**Table 3:** Risk reduction of hospitalisation after full vaccination with booster dose vs full vaccination 6 months or more before positive test in COVID-19 cases by age, sex and underlying conditions, Navarre, Spain, 3 January–20 March 2022 (n = 28,867)

Study group by vaccination status	Crude analysis		Adjusted analysis^a^	
	OR	95% CI	OR	95% CI
**All cases **				
Fully vaccinated ≥ 6 months before positive test	Reference		Reference	
Fully vaccinated + booster dose	2.00	1.57–2.55	0.38	0.28–0.52
**Aged 18–64 years **				
Fully vaccinated ≥ 6 months before positive test	Reference		Reference	
Fully vaccinated + booster dose	1.00	0.64–1.57	0.95	0.58–1.53
**Aged ≥ 65 years **				
Fully vaccinated ≥ 6 months before positive test	Reference		Reference	
Fully vaccinated + booster dose	0.23	0.17–0.32	0.22	0.16–0.32
**Men **				
Fully vaccinated ≥ 6 months before positive test	Reference		Reference	
Fully vaccinated + booster dose	2.47	1.77–3.44	0.48	0.31–0.74
**Women **				
Fully vaccinated ≥ 6 months before positive test	Reference		Reference	
Fully vaccinated + booster dose	1.61	1.13–2.28	0.30	0.19–0.47
**Cases with underlying conditions **				
Fully vaccinated ≥ 6 months before positive test	Reference		Reference	
Fully vaccinated + booster dose	1.41	1.08–1.85	0.40	0.29–0.57
**Cases without underlying conditions **				
Fully vaccinated ≥ 6 months before positive test	Reference		Reference	
Fully vaccinated + booster dose	1.25	0.70–2.21	0.28	0.13–0.60

CI: confidence interval; COVID-19: coronavirus disease; OR: odds ratio.

^a^ OR adjusted for age group (18–44, 45–64, 65–74, 75–84 and ≥ 85 years), sex, underlying conditions (immunocompromised, other major chronic conditions and none), and month.

### Risk reduction of severe disease in vaccinated COVID-19 cases

The proportion of severe disease was 1.1% among unvaccinated COVID-19 cases and 10.2% among unvaccinated cases aged 65 and older. In the overall analysis of COVID-19 cases, full vaccination reduced considerably the risk of severe disease during the first 6 months (aOR: 0.13; 95% CI: 0.06–0.28) and after the sixth month (aOR: 0.18; 95% CI: 0.10–0.31; p_comparison_ = 0.432), as well as after a booster dose (aOR: 0.06; 95% CI: 0.04–0.09). Full vaccination significantly reduced the risk of severe disease regardless of the sex, age and underlying conditions (Table 4).

**Table 4:** Risk reduction of severe disease in vaccinated COVID-19 cases vs unvaccinated cases by age, sex and underlying conditions, Navarre, Spain, 3 January–20 March 2022 (n = 58,952)

Study group and vaccination status	COVID-19 cases			Crude analysis		Adjusted analysis^ b^	
	All	Severe^a^		OR	95% CI	OR	95% CI
	n	n	%				
**All cases**							
Unvaccinated	3,873	41	1.1	Reference		Reference	
Partially vaccinated	550	2	0.4	0.34	0.08–1.41	0.73	0.17–3.17
Fully vaccinated < 6 months before positive test	25,662	9	0.0	0.03	0.02–0.07	0.13	0.06–0.28
Fully vaccinated ≥ 6 months before positive test	10,467	21	0.2	0.19	0.11–0.32	0.18	0.10–0.31
Fully vaccinated + booster dose	18,400	83	0.5	0.42	0.29–0.62	0.06	0.04–0.09
**Aged 18–64 years**							
Unvaccinated	3,578	11	0.3	Reference		Reference	
Partially vaccinated	529	0	0.0	NA		NA	
Fully vaccinated < 6 months before positive test	25,453	5	0.0	0.06	0.02–0.18	0.08	0.03–0.23
Fully vaccinated ≥ 6 months before positive test	10,069	7	0.1	0.23	0.09–0.58	0.12	0.05–0.31
Fully vaccinated + booster dose	9,807	8	0.1	0.27	0.11–0.66	0.16	0.06–0.41
**Aged ≥ 65 years**							
Unvaccinated	295	30	10.2	Reference		Reference	
Partially vaccinated	21	2	9.5	0.93	0.21–4.19	1.51	0.31–7.31
Fully vaccinated < 6 months before positive test	209	4	1.9	0.17	0.06–0.50	0.28	0.09–0.83
Fully vaccinated ≥ 6 months before positive test	398	14	3.5	0.32	0.17–0.62	0.23	0.12–0.46
Fully vaccinated + booster dose	8,593	75	0.9	0.08	0.05–0.12	0.06	0.04–0.10
**Men **							
Unvaccinated	1,820	22	1.2	Reference		Reference	
Partially vaccinated	257	1	0.4	0.32	0.04–2.38	0.72	0.09–5.65
Fully vaccinated < 6 months before positive test	12,448	5	0.0	0.03	0.01–0.09	0.12	0.04–0.33
Fully vaccinated ≥ 6 months before positive test	4,878	13	0.3	0.22	0.11–0.43	0.19	0.09–0.40
Fully vaccinated + booster dose	7,919	49	0.6	0.51	0.31–0.84	0.06	0.04–0.11
**Women **							
Unvaccinated	2,053	19	0.9	Reference		Reference	
Partially vaccinated	293	1	0.3	0.37	0.05–2.75	0.70	0.08–5.87
Fully vaccinated < 6 months before positive test	13,214	4	0.0	0.03	0.01–0.10	0.15	0.05–0.50
Fully vaccinated ≥ 6 months before positive test	5,589	8	0.1	0.15	0.07–0.35	0.16	0.07–0.38
Fully vaccinated + booster dose	10,481	34	0.3	0.35	0.20–0.61	0.06	0.03–0.11
**Cases with underlying conditions **							
Unvaccinated	930	29	3.1	Reference		Reference	
Partially vaccinated	113	2	1.8	0.56	0.13–2.38	1.21	0.26–5.55
Fully vaccinated < 6 months before positive test	5,280	8	0.2	0.05	0.02–0.10	0.22	0.09–0.51
Fully vaccinated ≥ 6 months before positive test	3,002	17	0.6	0.18	0.10–0.32	0.21	0.11–0.40
Fully vaccinated + booster dose	8,628	75	0.9	0.27	0.18–0.42	0.08	0.05–0.12
**Cases without underlying conditions **							
Unvaccinated	2,943	12	0.4	Reference		Reference	
Partially vaccinated	437	0	0.0	NA		NA	
Fully vaccinated < 6 months before positive test	20,382	1	0.0	0.01	0.00–0.09	0.03	0.00–0.21
Fully vaccinated ≥ 6 months before positive test	7,465	4	0.1	0.13	0.04–0.41	0.12	0.04–0.40
Fully vaccinated + booster dose	9,772	8	0.1	0.20	0.08–0.49	0.03	0.01–0.08

CI: confidence interval; COVID-19: coronavirus disease; NA: not available: OR: odds ratio.

^a^ Severe cases were confirmed cases who were admitted to the intensive care unit or who died because of COVID-19.

^b^ OR adjusted for age (18–44, 45–64, 65–74, 75–84 and ≥ 85 years), sex, underlying conditions (immunocompromised, other major chronic conditions and none), and month.

A person was considered ‘fully vaccinated’ after receiving one dose of Janssen vaccine or two doses of Comirnaty, Spikevax or Vaxzevria, and ‘partially vaccinated’ if only one of the two recommended doses of COVID-19 vaccine received.

Compared with cases fully vaccinated 6 months or more before a positive test, those who had received a booster dose had lower risk of severe disease (aOR: 0.38; 95% CI: 0.21–0.68) overall and in most analysed categories. However, a booster dose did not significantly reduce the risk of severe disease in cases aged 18–64 years (Table 5).

**Table 5:** Risk reduction of severe disease^a^ of full vaccination with booster dose vs full vaccination 6 months or more before positive test in COVID-19 cases by age, sex and underlying conditions, Navarre, Spain, 3 January–20 March 2022 (n = 28,867)

Study group and vaccination status	Crude analysis			Adjusted analysis^b^			
	OR	95% CI		OR		95% CI	
**All cases **							
Fully vaccinated ≥ 6 months before positive test	Reference			Reference			
Fully vaccinated + booster dose	2.25		1.40–3.64	0.38			0.21–0.68
**Aged 18–64 years **							
Fully vaccinated ≥ 6 months before positive test	Reference			Reference			
Fully vaccinated + booster dose	1.17		0.43–3.24	1.28	0.45–3.66		
**Aged ≥ 65 years **							
Fully vaccinated ≥ 6 months before positive test	Reference			Reference			
Fully vaccinated + booster dose	0.24		0.14–0.43	0.25	0.14–0.46		
**Men **							
Fully vaccinated ≥ 6 months before positive test	Reference			Reference			
Fully vaccinated + booster dose	2.33		1.26–4.30	0.31	0.14–0.65		
**Women **							
Fully vaccinated ≥ 6 months before positive test	Reference			Reference			
Fully vaccinated + booster dose	2.27		1.05–4.91	0.52	0.21–1.31		
**Cases with underlying conditions**							
Fully vaccinated ≥ 6 months before positive test	Reference			Reference			
Fully vaccinated + booster dose	1.54		0.91–2.61	0.41	0.22–0.78		
**Cases without underlying conditions **							
Fully vaccinated ≥ 6 months before positive test	Reference			Reference			
Fully vaccinated + booster dose	1.53		0.46–5.08	0.21	0.05–0.97		

CI: confidence interval; COVID-19: coronavirus disease; OR: odds ratio.

^a^ Severe cases were confirmed cases who were admitted to the intensive care unit or who died due to COVID-19.

^b^ OR adjusted for age group (18–44, 45–64, 65–74, 75–84 and ≥ 85 years), sex, underlying conditions (immunocompromised, other major chronic conditions and none), and month.

### Sensitivity analysis

Sensitivity analyses limited to weeks with more than 90% of cases caused by the SARS-CoV-2 Omicron BA.1 subvariant or excluding self-testing results, shown in Supplementary Tables S2 and S3 respectively, provide similar estimates of the risk reduction of hospitalisation and severe disease in vaccinated COVID-19 cases.

## Discussion

In many countries within Europe and globally, the highest incidence of COVID-19 cases was reached during the SARS-CoV-2 Omicron wave in early 2022 [2,3]. Our results show that a full primary course of COVID-19 vaccination substantially reduced the risk of hospitalisation (94%) and severe outcomes (87%) during the Omicron BA.1-predominant period. In this context, risk reduction of hospitalisation and severe disease among the large number of COVID-19 cases caused by the Omicron subvariant likely had a very important impact in the health of the population. 

In fully vaccinated COVID-19 cases, the reduction of the risk of hospitalisation was lower (84% vs 94%) when infection occurred 6 months or more after vaccination. In people over 65 years of age, a booster dose was effective to regain protection against risk of hospitalisation and severe disease, which supports the recommendation of the booster dose in older or immunocompromised people [13,14,21].

Previous studies have suggested that the COVID-19 vaccine effectiveness was reduced and the effect attenuated over time to a stronger degree during the Omicron variant period versus the Delta variant period [14-17,22]. Moreover, a booster dose of COVID-19 vaccine was shown to be more relevant to maintain protection against disease caused by the Omicron variant [13-15]. Our results are aligned with other studies that have evaluated COVID-19 vaccine effectiveness in preventing COVID-19 hospitalisation and severe outcomes during the Omicron period [23-26]. The findings of our study were consistent regardless of whether or not self-test results were included.

The slight decrease in risk reduction of hospitalisation and severe disease by COVID-19 vaccination observed 6 months after full vaccination could be related to waning immunity, although a possible bias cannot be ruled out entirely [27]. In cases aged 18–64 years, the booster dose did not show greater risk reduction of hospitalisation or severe disease compared with full vaccination 6 months or more before positive test. This could be explained in part because a booster dose was initially offered only to immunocompromised subjects who had completed the primary scheme earlier [27]. However, these results also suggest that the booster dose was additionally protective in people over 65 years of age, but was not yet relevant in immunocompetent adults aged under 65 years, most of whom had completed vaccination less than 1 year before [5].

The present study has advantages given the case-to-case comparison of COVID-19 cases that were obtained from the same information source, which ensures the comparability of data. The enhanced surveillance covered all COVID-19 cases confirmed in the region, and during the study period, there was high availability of diagnostic tests and of personnel for testing of all suspected cases, and only hospitalisations, ICU admissions and deaths related to COVID-19 according to a medical revision were considered. Our results may be of interest to other countries, since the variant distribution observed was similar to that described in Europe [23-25].

This study has some limitations. Our results are not estimates of the vaccine effectiveness in preventing hospitalisations and severe disease in the population but among COVID-19 cases, and the vaccine effect in preventing SARS-CoV-2 infections was not considered [28]. Vaccination coverage varied greatly with age, comorbidity and month, but analyses were adjusted for these variables. Only cases in the resident population were considered to avoid incomplete medical information. Healthcare workers and nursing home residents were excluded, so our results would not be applicable to these groups. The small number of immunocompromised cases limited a specific analysis of this group. Since cases with known previous COVID-19 were excluded, we cannot make conclusions about the preventive role of prior infections that has been suggested by other authors [11,29]. The vaccine effect may be different by vaccine brand [30], but this was not considered in the present study. The SARS-CoV-2 variant was only assessed in a small sample of cases with a positive PCR result, which included a higher proportion of hospitalised and severe cases. Nevertheless, the high proportion of the Omicron variant in this small sample was consistent with reports of other countries. Most cases studied were diagnosed during the period when the Omicron BA.1 subvariant was predominant, therefore the results may not be valid for other Omicron subvariants. Most booster doses had been recently received (ca within 5 months), which impaired evaluating the long-term risk reduction.

## Conclusions

Full COVID-19 vaccination significantly reduces the risk of hospitalisation and severe outcomes in COVID-19 cases caused by the Omicron variant, and a booster dose notably improves this effect as compared with that observed 6 months or more after full vaccination in those aged 65 years and older. Given the reduced effectiveness of the COVID-19 vaccines in preventing infection by the Omicron variant and its wide circulation globally during 2022 that continues into the beginning of 2023, full COVID-19 vaccination of the population and timely administration of booster doses are essential in reducing the incidence of COVID-19 severe outcomes.

## Supplementary Data

207000 Supplement
