# Supplementary material for: Risk reduction of hospitalisation and severe disease in vaccinated COVID-19 cases during the SARS-CoV-2 variant Omicron BA.1-predominant period, Navarre, Spain, January to March 2022
Source: Euro Surveill. 2023 Feb 2;28(5):2200337. doi: 10.2807/1560-7917.ES.2023.28.5.2200337 (PMC9896606; doi:10.2807/1560-7917.ES.2023.28.5.2200337)
Supplement: Supplement [file 22-00337_CASTILLA_SUPPLEMENT.pdf]

SUPPLEMENTARY MATERIAL

This supplementary material is hosted by *Eurosurveillance* as supporting information alongside the article "Risk reduction of hospitalisation and severe disease in vaccinated COVID-19 cases during the Omicron BA.1 predominant period, Navarre, Spain, January to March 2022" on behalf of the authors who remain responsible for the accuracy and appropriateness of the content. The same standards for ethics, copyright, attributions and permissions as for the article apply. Supplements are not edited by *Eurosurveillance* and the journal is not responsible for the maintenance of any links or email addresses provided therein.

Supplementary Table S1. Characteristics of the study population of COVID-19 cases by vaccination status. Navarre, Spain, 3 January to 20 March 2022 (n = 58,402)[a]

	**Unvaccinated**		**Fully vaccinated <6 months before positive test**		**Fully vaccinated ≥6 months before positive test**		**Fully vaccinated + additional dose**	
	n	%	n	%	n	%	n	%
**Age groups, years**								
18 – 44	2428	62.7	20,091	78.3	3119	29.8	3145	17.1
45 – 64	1150	29.7	5362	20.9	6950	66.4	6662	36.2
65 – 74	173	4.5	178	0.7	192	1.8	4238	23.0
75 – 84	74	1.9	21	0.1	120	1.2	2813	15.3
≥ 85	48	1.2	10	0.0	86	0.8	1542	8.4
**Sex**								
Male	1820	47.0	12,448	48.5	4878	46.6	7919	43.0
Female	2053	53.0	13,214	51.5	5589	53.4	10,481	57.0
**Underlying conditions**								
Immunocompromised	35	0.9	147	0.6	86	0.8	232	1.3
Other major chronic conditions	895	23.1	5133	20.0	2916	27.9	8396	45.6
None	2943	76.0	20,382	79.4	7465	71.3	9772	53.1
**Hospitalised**								
No	3723	96.1	25,637	99.9	10,381	99.2	18,100	98.4
Yes	150	3.9	25	0.1	86	0.8	300	1.6
**Severe disease** [b]								
No	3832	98.9	25,653	100.0	10,446	99.8	18,317	99.5
Yes	41	1.1	9	0.0	21	0.2	83	0.5
**Admitted to the intensive care unit**								
No	3858	99.6	25,657	100.0	10,461	99.9	18,388	99.9
Yes	15	0.4	5	0.0	6	0.1	12	0.1
**Death**								
No	3843	99.2	25,658	100.0	10,450	99.8	18,327	99.6
Yes	30	0.8	4	0.0	17	0.2	73	0.4
**Month**								
January	3117	80.5	23,431	91.3	8476	81.0	11,077	60.2
February	550	14.2	2010	7.8	1343	12.8	4416	24.0
March	206	5.3	221	0.9	648	6.2	2907	15.8
**Period**								
3 Jan-20 Feb (>90% of BA.1)	3592	92.7	25,278	98.5	9575	91.5	14,649	79.6
21 Feb-20 Mar (mix of BA.1 and BA.2)	281	7.3	384	1.5	892	8.5	3751	20.4
**Type of test**								
RT-qPCR	522	13.5	1513	5.9	723	6.9	1896	10.3
Antigent test	3190	82.4	23,296	90.8	9232	88.2	13,912	75.6
Antigen self-testing	161	4.2	853	3.3	512	4.9	2592	14.1
**Total**	3873	100	25,662	100	10,467	100	18,400	100

[a] Cases partially vaccinated are not included in this table.
[b] Severe disease includes confirmed cases who were admitted to intensive care unit or who died due to COVID-19.

Supplementary Table S2. Sensitivity analyses of risk reduction of hospitalisation and severe disease in vaccinated COVID-19 cases in weeks with more than 90% of cases due to the SARS-CoV-2 Omicron BA.1 subvariant. Navarre, Spain, 3 January to 20 March 2020 (n = 53,580)

	All cases	Hospitalised or severe COVID-19 cases[a]		Crude odds ratio (95% CI)	Adjusted odds ratio (95% CI) [b]
	n	n	%		
**Risk reduction of hospitalisation**					
Unvaccinated	3592	147	4.1	1	1
Partially vaccinated	523	3	0.6	0.14 (0.04-0.43)	0.20 (0.06-0.65)
Fully vaccinated <6 months before positive test	25,278	23	0.1	0.02 (0.01-0.03)	0.05 (0.03-0.08) [c]
Fully vaccinated ≥6 months before positive test	9575	81	0.8	0.20 (0.15-0.26)	0.15 (0.11-0.20) [c,d]
Fully vaccinated + booster dose	14,649	269	1.8	0.44 (0.36-0.54)	0.05 (0.04-0.07) [d]
**Risk reduction of severe disease**					
Unvaccinated	3592	41	1.1	1	1
Partially vaccinated	523	2	0.4	0.33 (0.08-1.38)	0.74 (0.17-3.24)
Fully vaccinated <6 months before positive test	25,278	8	0.0	0.03 (0.01-0.06)	0.11 (0.05-0.24)
Fully vaccinated ≥6 months before positive test	9575	21	0.2	0.19 (0.11-0.32)	0.17 (0.10-0.30) [d]
Fully vaccinated + bosster dose	14,649	77	0.5	0.46 (0.31-0.67)	0.06 (0.04-0.09) [d]

CI: confidence interval.
[a] Severe cases included confirmed cases who were admitted to intensive care unit or who died due to COVID-19.
[b] Odds ratio adjusted for age (18-44, 45-64, 65-74, 75-84 and ≥85 years), sex, underlying conditions (immunocompromised, other major chronic conditions and none), and month.
[c] Comparison between fully vaccinated cases ≥6 months and <6 months before positive test, p-value<0.05.
[d] Comparison of cases fully vaccinated plus additional dose versus fully vaccinated cases ≥6 months before positive test, p-value<0.001.

Supplementary Table S3. Sensitivity analyses of risk reduction of hospitalisation or severe disease in vaccinated COVID-19 cases including RT-qPCR or antigen test results and excluding self-testing results. Navarre, Spain, 3 January to 20 March 2022 (n = 54,811)

	**All cases**	**Hospitalised or severe COVID-19 cases**[a]		**Crude odds ratio (95% CI)**	**Adjusted odds ratio (95% CI)** [b]
	**n**	**n**	**%**		
**Risk reduction of hospitalisation**					
Unvaccinated	3712	148	4.0	1	1
Partially vaccinated	527	4	0.9	0.18 (0.07-0.50)	0.26 (0.09-0.73)
Fully vaccinated <6 months before positive test	24,809	25	0.1	0.02 (0.02-0.04)	0.06 (0.04-0.09) [c]
Fully vaccinated ≥6 months before positive test	9955	85	0.9	0.21 (0.16-0.27)	0.15 (0.11-0.21) [c,d]
Fully vaccinated + booster dose	15,808	290	1.8	0.45 (0.37-0.55)	0.06 (0.04-0.07) [d]
**Risk reduction of severe disease**					
Unvaccinated	3712	40	1.1	1	1
Partially vaccinated	527	2	0.4	0.35 (0.08-1.45)	0.73 (0.17-3.18)
Fully vaccinated <6 months before positive test	24,809	9	0.0	0.03 (0.02-0.07)	0.13 (0.06-0.29)
Fully vaccinated ≥6 months before positive test	9955	21	0.2	0.19 (0.11-0.33)	0.18 (0.10-0.32) [d]
Fully vaccinated + booster dose	15,808	80	0.5	0.47 (0.32-0.69)	0.06 (0.04-0.10) [d]

CI: confidence interval.
[a] Severe cases included confirmed cases who were admitted to intensive care unit or who died due to COVID-19.
[b] Odds ratio adjusted for age (18-44, 45-64, 65-74, 75-84 and ≥85 years), sex, underlying conditions (immunocompromised, other major chronic conditions and none), and month.
[c] Comparison between fully vaccinated cases ≥6 months and <6 months before positive test, p-value<0.05.
[d] Comparison of cases fully vaccinated plus additional dose versus fully vaccinated cases ≥6 months before positive test, p-value<0.001.
